# Supplementary material for: Voltage control of magnetic anisotropy in epitaxial Ru/Co2FeAl/MgO heterostructures
Source: Sci Rep. 2017 Mar 23;7:45026. doi: 10.1038/srep45026 (PMC5362931; doi:10.1038/srep45026)
Supplement: Supplementary Information [file srep45026-s1.pdf]

Supplementary Information

Voltage control of magnetic anisotropy in epitaxial Ru/Co_2FeAl/MgO heterostructures

Zhenchao Wen,[1] Hiroaki Sukegawa,[1] Takeshi Seki,[2,3] Takahide Kubota,[2,3] Koki Takanashi,[2,3] and Seiji Mitani[*1,4]

[1]*National Institute for Materials Science (NIMS), Tsukuba 305-0047, Japan*
[2]*Institute for Materials Research (IMR), Tohoku University, Sendai 980-8577, Japan*
[3]*Center for Spintronics Research Network (CSRN), Tohoku University, Sendai 980-8577, Japan*
[4]*Gradate School of Pure and Applied Sciences, University of Tsukuba, 305-8577, Japan*

*Email: Mitani.Seiji@nims.go.jp

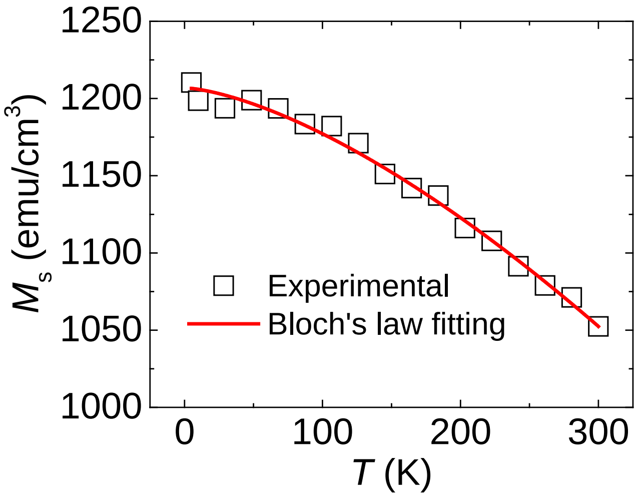

Figure S1. Temperature dependence of saturation magnetization M_s for the Co_2FeAl (CFA) layer in the Ru/CFA/MgO heterostructure. The measurement of M_s at low temperatures was performed using a superconducting quantum interference device (SQUID) magnetometer. The M_s-T curve was well fitted by the Bloch's law with the Curie temperature T_c = 1181 K and M_s (0 K) = 1206 emu/cm^3.
